# Preoperative marking of a submillimeter metastatic pulmonary tumor using a mobile computed tomography scan with a navigation system: A case report

**DOI:** 10.1016/j.ijscr.2021.01.021

**Published:** 2021-01-18

**Authors:** Asuka Uebayashi, Ryo Fujikawa, Yoshifumi Arai, Toru Nakamura, Kazuhito Funai

**Affiliations:** a Department of General Thoracic Surgery, Seirei Hamamatsu General Hospital, 2-12-12 Sumiyoshi, Nakaku, Hamamatsu-city, Shizuoka 430-8558, Japan; b Department of Pathology, Seirei Hamamatsu General Hospital, 2-12-12 Sumiyoshi, Nakaku, Hamamatsu-city, Shizuoka 430-8558, Japan; c First Department of Surgery, Hamamatsu University School of Medicine, 1-20-1 Handayama, Higashi-ku, Hamamatsu City, Shizuoka 431-3192, Japan

**Keywords:** CT, computed tomography, VATS, video assisted thoracic surgery, Image-guided video assisted thoracic surgery (iVATS), Mobile computed tomography, Navigation system, Non-palpable lung nodule, Preoperative marking, Case report

## Abstract

**Introduction and importance:**

Preoperative localization of non-palpable lung nodules plays an important role in video assisted thoracic surgery (VATS). Although percutaneous computed tomography (CT)-guided hook wire marking has become widely accepted, it is accompanied by rare but fatal complications such as air embolisms. We herein report a case of a submillimeter pulmonary nodule successfully localized by a mobile CT scan with a navigation system.

**Case presentation:**

A 40-year-old-man presented with the two right pulmonary nodules 4 years after a radical left nephrectomy for a renal clear cell carcinoma. One of the nodules was too small to palpate and preoperative marking was applied using a mobile CT scan with a navigation system. We successfully performed VATS wedge resection for both nodules and confirmed a pathological diagnosis of a metastasis from the renal cell carcinoma. The maximum pathological size of the smaller nodule was 500 μm.

**Clinical discussion:**

Preoperative marking of the lower lobe lesion in the present case was essential for VATS. Our novel technique was helpful for the precise marking without any morbidity.

**Conclusion:**

Preoperative marking using a mobile CT scan with a navigation system is safe and easily applicable. It might be a useful option for VATS of non-palpable lung nodules.

## Highlights

• A submillimeter metastatic lung tumor was resected successfully by intraoperative marking using a mobile CT with a navigation system.
• This method is safer and might be more accurate than the traditional hook wire marking without the necessity of percutaneous lung puncture.
• It also reduced a patient’s stress because the whole procedure could be done at a single stage under general anesthesia.

## 1 Background

Detection of small lung nodules has increased with the development of diagnostic technologies such as high-resolution computed tomography (CT) and preoperative localization of non-palpable lesions is essential for video assisted thoracic surgery (VATS). Although percutaneous CT-guided hook wire marking has become widely accepted [[1], [2], [3], [4], [5]], it requires percutaneous pleural puncture and is accompanied by rare but fatal complications such as air embolisms [6,7]. We herein report a case of a nonpalpable pulmonary nodule successfully resected using a novel mobile CT scan with a navigation system for preoperative marking. This work has been reported in line with the SCARE criteria [8].

## 2 Case presentation

**Fig. 1:**
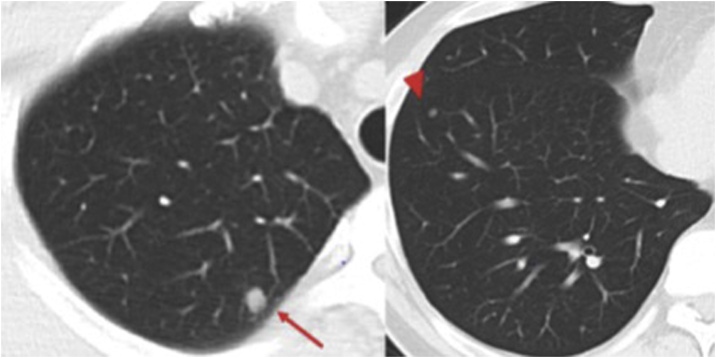
Chest computed tomography showing pulmonary nodules in the right upper (arrow) and lower lobes (arrowhead).

A 40-year-old-man presented with right pulmonary nodules 4 years after a radical left nephrectomy for a renal clear cell carcinoma (pT2,ly0,v0,pStageⅡ:UICC8th. Fuhrman grade 2) without any drug use nor allergic history. He had no significant familial history. Chest CT revealed two circumscribed nodules in the right upper and lower lobes respectively, suggesting metastatic lung tumors (Fig. 1). Without any metastatic lesion, he was planned to undergo a surgical resection of both nodules. The lower lobe nodule was deemed non-palpable and we applied intraoperative marking using a mobile CT scan with a navigation system.

**Fig. 2:**
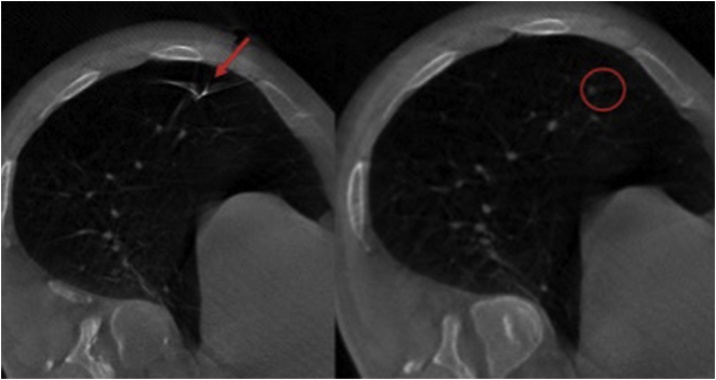
The second scan showing the needle (arrow) placed 7 mm caudally from the target nodule in the lower lobe (open circle).

The details of the procedure have been described elsewhere [9]. Briefly, the patient was placed in the left decubitus position with a navigation reference fixed on the anterior superior iliac spine under general anesthesia. A mobile CT: O-arm™ (Medtronic Japan Co. Ltd Tokyo, Japan) was then brought over the patient to obtain three-dimensional (3D) images. After the initial scanning, a navigation system: Stealth Station™S7 (Medtronic Japan Co. Ltd Tokyo, Japan) synchronized those images with the hand probe through the navigation reference on the patient. A skin marking was placed at the closest point over the target lesion by using the hand probe as virtual fluoroscopy. We marked the lung surface just beneath the skin marking by using the dye stamping method [10]. We anchored a needle (4-0 PDS, PS-4) on the lung dye marking and confirmed the proper location of the needle and target nodule by a second CT scan (Fig. 2). After ligating the thread of the needle, we performed a wedge resection of the marked lung with an enough margin of 15 mm. The time duration between the initial scanning and the removal of the target lung was 75 min. Cost of the scanning is equivalent to that of the conventional CT.

The nodule in the upper lobe was resected in the usual manner. The operative time was 118 min without any procedure-related morbidity.

**Fig. 3:**
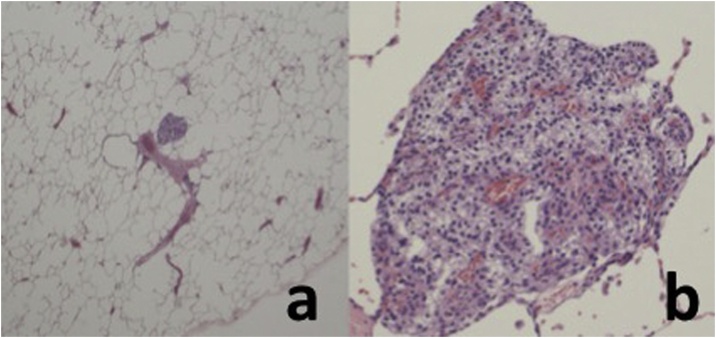
Pathological specimen of the nodule in the lower lobe showing the cytoplasm filled with glycogen and phospholipids surrounded by a vascular connective wave, consistent with metastasis from a renal clear cell carcinoma (Hematoxylin-Eosin stain, a: ×40, b: ×200).

The histopathological examination of both specimens revealed that the cytoplasm was filled with glycogen and phospholipids, consistent with a metastasis from the renal clear cell carcinoma (Fig. 3). The maximum pathological size of the nodule in the lower lobe was 500 μm. The surgical margin status was negative for malignancy. He is currently disease free at 26 months after the surgery.

## 3 Discussion

The lungs are one of the most frequent metastatic sites from a renal cell carcinoma and a pulmonary metastasectomy is a feasible option in selected patients [11]. Two favorable prognostic factors proposed in the literature were met for the present case, the achievement of a complete resection [[12], [13], [14], [15]] and a small diameter of the lesion [[14], [15], [16], [17]]. Our case had only two metastases with a maximum diameter of 8 mm. The lower lobe lesion was too small to palpate and therefore preoperative marking was essential for a VATS resection.

Various methods have been proposed for the localization of non-palpable small pulmonary nodules. A CT-guided hook wire marking is one of the most common procedures and is often accompanied by procedure-related complications such as a pneumothorax and pulmonary hemorrhage [[1], [2], [3], [4]]. Air embolisms rarely occur, but could be fatal [6]. These morbidities are all derived from a percutaneous pleural puncture itself and inevitable as long as the hook wire marking is applied. To avoid those morbidities, we introduced a mobile CT with a navigation system, which had been widely used in spinal and orthopedic surgeries [18,19].

The O-arm™ is a mobile preoperative CT imaging system. The obtained images are transferred to the Stealth Station™S7 navigation system and allow virtual fluoroscopy to identify the preferred skin marking site [20]. The second CT scanning after the lung marking contributes to the verification of the positional relationship between the needle and target lesion without the necessity for digital palpation. A redo marking is also available for marking failure cases with this method [9]. In the present case, we could localize the submillimeter nodule without any procedural related morbidity. Because both the marking procedure and surgery were completed under general anesthesia in the operating room as a single stage procedure, it would be beneficial to reduce the patients’ stress as compared to the traditional marking method. These results suggest that this method would be a feasible option for image-guided VATS for preoperative marking of non-palpable lung nodules especially in the peripheral region [9].

## 4 Conclusion

We herein report a case of a submillimeter metastatic lung tumor successfully resected using a mobile CT with a navigation system for the preoperative marking. This method is safe and easily applicable and might be a useful option for VATS of non-palpable lung nodules even in submillimeter cases.　

## Declaration of Competing Interest

The authors declare that they have no competing interests.

## Funding

Not applicable.

## Ethical approval

Not applicable.

## Consent

Written informed consent was obtained from the patient for publication of this case report and accompanying images. A copy of the written consent is available for review by the Editor-in-Chief of this journal on request.

## Author contribution

Asuka Uebayashi wrote this paper. All authors read and approved the final manuscript.

## Registration of research studies

Not applicable.

## Guarantor

Toru Nakamura.

## Provenance and peer review

Not commissioned, externally peer-reviewed.
